# Detection and characterization of micro-organisms linked to unsealed drugs sold in Ihiagwa community, Owerri, Imo State, Nigeria

**DOI:** 10.1099/acmi.0.000752.v3

**Published:** 2024-02-16

**Authors:** Uzochukwu G. Ekeleme, Vivian O. Ikwuagwu, Uchechukwu M. Chukwuocha, Jane C. Nwakanma, Stephanie A. Adiruo, Ikenna O. Ogini, Ifeoma U. Ude

**Affiliations:** ^1^​ Department of Public Health, Federal University of Technology, Owerri, Imo State, Nigeria; ^2^​ Department of Microbiology, Gregory University Uturu, Abia State, Nigeria; ^3^​ Department of Health Education, Alvan Ikoku Federal College of Education, Owerri, Nigeria

**Keywords:** detection, characterization, micro-organisms, unsealed drugs

## Abstract

The contamination of pharmaceutical products by micro-organisms poses a significant risk to public health. This study was conducted to detect and characterize micro-organisms associated with unsealed drugs sold in Ihiagwa community in Owerri, Imo State, Nigeria. A variety of microbiological techniques were employed to analyse samples from unsealed drug containers. The identification process involved morphological, biochemical and sugar utilization methods, aiding in the accurate determination of microbial species. Microbial contamination was observed in 42 (84 %) out of 50 samples, with contaminants including bacteria and fungi. The range of contamination is between 1.2±0.01×10^3^ and 2.3±0.02×10^3^ c.f.u. ml^−1^ for viable count, 0.1±0.02×10^2^ and 0.3±0.01×10^2^ c.f.u. g^−1^ for coliform count and 0.2±0.01×10^1^ and 0.5±0.01×10^1^ c.f.u. g^−1^ for fungi count. The identified microbes were *Staphylococcus aureus*, *Escherichia coli*, *Pseudomonas aeruginosa*, *Candida albicans* and *Aspergillus niger*. The most common bacterial isolate was *S. aureus* (51.8 %), while *C. albicans* (73.3 %) was the most prevalent fungus. Among the pharmacies and healthcare facilities examined, the Uchems pharmacy had the highest proportion of bacterial isolates (37 %), followed by the Stepwise pharmacy (22.2 %), while the lowest proportion was found at the Roseline Health Clinic (7.4 %). The identification of potentially harmful micro-organisms in these unsealed drug container samples emphasizes the importance of stringent quality control measures and improved handling, storage and packaging practices to ensure product safety and efficacy, especially among pharmacetical dealers.

## Data Summary

All data generated or analysed during this study are included in the published article.

## Introduction

### Background to the study

Pharmaceutical products, including both prescription and over-the-counter drugs, are expected to meet stringent quality standards to ensure their safety and efficacy. The presence of micro-organisms, such as bacteria, fungi and viruses, in pharmaceutical products can compromise their quality and pose health risks to consumers [1]. Microbial contamination can occur at any stage of the drug manufacturing, distribution, or dispensing process [2]. In the case of unsealed drugs sold in informal settings such as street markets, the risk of contamination is particularly high due to the lack of proper storage conditions and quality control measures.

Previous studies have highlighted the prevalence of unsealed drugs in various regions and the associated risks. In a study conducted by Smith *et al*. [3], it was found that a significant proportion of drugs sold in low-resource settings were unsealed or had damaged packaging, increasing the likelihood of contamination. Moreover, research by Johnson *et al*. [4] demonstrated that unsealed drugs were more susceptible to microbial contamination, with potential health implications for consumers. Access to safe and effective pharmaceuticals is fundamental to public health, ensuring that individuals receive the intended therapeutic benefits of medications while avoiding harm from contaminants or substandard products [5]. However, in many communities around the world, including Ihiagwa community in Owerri, Imo State, Nigeria, the sale and consumption of unsealed drugs pose significant health risks. Unsealed drugs are pharmaceutical products that have been tampered with, improperly stored, or lack the necessary packaging and labelling that ensure their authenticity and safety.

Ihiagwa community, like many other areas, faces challenges related to the availability and quality of pharmaceuticals. The unregulated sale of unsealed drugs in local markets and by informal vendors has become a pervasive issue, exposing community members to potential health hazards. Unsealed drugs can be compromised in various ways, including exposure to environmental contaminants, improper storage conditions and even intentional adulteration. Such drugs may not only be ineffective in treating illnesses but can also introduce harmful micro-organisms into the body, leading to infections and adverse health outcomes.

Microbial contamination of pharmaceutical products is a well-documented concern globally. Pathogenic bacteria, fungi and other micro-organisms can thrive in improperly stored or handled drugs, rendering them unsafe for consumption [6]. The ingestion or application of contaminated drugs can result in a range of health complications, especially among vulnerable populations, such as children, the elderly and individuals with weakened immune systems [7].

Addressing the issue of unsealed drugs and their potential microbial contamination in Ihiagwa community is of paramount importance for public health. However, comprehensive studies examining the extent and nature of this problem are limited. By undertaking this research, we aim to shed light on the microbial quality of pharmaceuticals sold in the community, thereby contributing to a safer and healthier environment for its residents.

### Rationale for the study

The isolation and identification of micro-organisms associated with unsealed drugs sold in Ihiagwa are of paramount importance as microbial contamination of pharmaceutical products can lead to severe health consequences, especially in immunocompromised individuals or those with preexisting medical conditions. Identifying the specific micro-organisms present in these unsealed drugs will help us assess to the potential health risks faced by consumers in the community.

Ensuring the quality of pharmaceutical products is a fundamental responsibility of regulatory agencies and healthcare providers. By investigating microbial contamination in unsealed drugs, this study contributes to the quality assurance efforts in the pharmaceutical sector and can guide future regulatory measures.

Consumers in this community, like those elsewhere, have the right to access safe and effective pharmaceutical products. Identifying and quantifying the microbial load in unsealed drugs will inform consumers about the risks associated with such products, enabling them to make informed decisions regarding their use.

### Objectives of the study

The primary objectives of this study are as follows:

1. Detect and identify micro-organisms present in unsealed drugs sold within Ihiagwa Community.
2. Characterize the types and prevalence of microbial contaminants in these drugs.
3. Provide evidence-based recommendations for improving pharmaceutical quality control and drug safety within the community.

## Methods

### Sample collection

The samples were collected from three registered and licensed practising pharmacies and the only two primary healthcare facilities in Ihiagwa, Owerri West L.G.A., Imo State, Nigeria.

The overall number of samples collected was 50. An average of 10 samples was collected from each of the three pharmacies known as Uchems, Silver and Stepwise. Additionally, 10 samples each were collected from the 2 primary healthcare facilities known as Ihiagwa Health Centre and Roseline Health Clinic. The samples were collected from unsealed drug containers of paracetamol, chloroquine and metronidazole at a ratio of 4 : 3 : 3, respectively. The drug containers of the pills were named Josedol for paracetamol (Sidom pharmaceutical company), chloroquine (Geneith Pharmaceutical Ltd) and Flanizol for metronidazole (See-best Pharm Chemists Ltd). The drug pills came in sealed containers and were opened later by personnel at the various local pharmacies and healthcare facilities. The pills were sold to people from the opened containers kept at room temperature.

Before sample collection, personal protective equipment (PPE; e.g. gloves and laboratory coats) was worn to maintain a sterile environment and protect from potential hazards.

Before using the Eswab, the integrity was checked to ensure that the packaging was intact and had not been compromised to maintain the sterility of the swab. The Eswab package was opened carefully without touching the swab’s sterile tip to avoid contamination, and the Eswab was held near the top of the handle to avoid touching the sterile swab tip.

The samples were collected aseptically using a sterile swab stick to avoid further external contamination during the collection process. This was done by using the Eswab to swab the entire circumference of the container to maximize the chances of detecting the potential contaminants, and the swab was placed back into the Eswab tube without touching the tube’s inner walls. Stuart transport medium (1 ml) was added into the Eswab tube to fully submerge the swab tip in the tube. The Eswab tube was tightly closed to prevent leakage and contamination. Each sample was labelled with the relevant information, such as the name of the sampled drug container, the location and the date of collection.

### Microbiological analysis

Microbiological analysis was carried out under strict aseptic conditions in the Laboratory of Infectious Disease and Molecular Epidemiology, Department of Public Health, Federal University of Technology, Owerri, Imo State, Nigeria.

The samples were first sorted in a biosafety cabinet II. The Eswab samples were vortexed, and 90 µl of eluate was used for culture inoculation per plate/test condition [8]. Each of the culture media [MacConkey agar with cefotaxime (Hardy), cetrimide agar (Hardy), mannitol salt agar, *Listeria* agar, *Salmonella–Shigella* agar, blood agar, chocolate agar and Sabouraud dextrose+chloramphenicol (Hardy)] received an inoculation of all the samples. Using an air incubator, plates were incubated at 37 °C for up to 48 h before being discarded if there was no growth and up to 7 days for Sabouraud dextrose with chloramphenicol [8]. All isolates recovered were stored at −80 °C in tryptic soy broth (TSB) with glycerol. Isolated micro-organisms were identified using a combination of morphological, biochemical and microscopic examinations of colony morphology, cell shape and size, which provided initial clues for identification. Biochemical assays such as the Gram stain, catalase test, oxidase test and sugar fermentation tests were performed to further classify the isolates [9].

## Results

In the realm of pharmaceutical safety and quality control, the isolation and identification of micro-organisms associated with unsealed drugs represent a critical endeavour. This study delves into the comprehensive results of our rigorous investigation, shedding light on the diverse microbial communities that may inhabit these unsealed drugs. Our findings are presented below, offering a comprehensive overview of the micro-organisms identified and their potential implications for drug safety and patient health.

The results showed that all of the unsealed sample drugs (tablets) containers were contaminated with micro-organisms (Table 1). The range of contamination was between 1.2±0.01×10^3^ and 2.3±0.02×10^3^ c.f.u. ml^−1^ for viable count, 0.1±0.02×10^2^ and 0.3±0.01×10^2^ c.f.u. g^−1^ for coliform count and 0.2±0.01×10^1^ and 0.5±0.01×10^1^ c.f.u. g^−1^ for fungi count.

**Table 1.** Total count of micro-organisms isolated from the unsealed drug (tablet) containers

Unsealed drugs	Total viable count (c.f.u. g^−1^) × 10^3^	Total coliform count (c.f.u. g^−1^) × 10^2^	Total fungi count (c.f.u. g^−1^) × 10^1^
Paracetamol	2.3±0.02	0.2±0.01	0.5±0.01
Chloroquine	1.2±0.01	0.1±0.02	0.2±0.01
Metronidazole	2.2±0.02	0.3±0.01	0.3±0.01

c.f.u, colony forming unit; g^−1^, per gram.

A total of 50 unsealed drug sample containers comprising paracetamol, chloroquine and metronidazole were analysed in this study. In the microbiological analysis microbial contamination was observed in 42 (84 %) out of 50 samples, with the contaminants including bacteria and fungi (Table 2).

**Table 2.** Types of micro-organisms isolated from unsealed drug containers (*n*=50)

Micro-organisms	No. of samples contaminated	Percentage (%) of samples contaminated
Bacteria	27	54.0
Fungi	15	30.0
Total	42	84.0

The morphological, biochemical and cultural characteristics of the micro-organisms isolated from the unsealed drug containers are represented in Tables 3 and 4. The identified microbes were *Staphylococcus aureus*, *Escherichia coli*, *Pseudomonas aeruginosa*, *Candida albicans* and *Aspergillus niger*.

**Table 3.** Identification of bacterial isolated from unsealed drug containers

	**Microscopy**				**Biochemical reactions**										**Carbohydrate Utilization**						
**Colony features**	Cell arrangement	Spore	Motility	Capsule	Catalase	Oxidase	Coagulase	Indole	Nitrate	MethylRed	VP	Urease	H_2_S	Citrate	Glucose	Sucrose	Lactose	maltose	manitol	xylose	**Organism**
Circular smooth colonies with light-yellow pigment on mannitol salt agar (MSA)	Gram-positive spherical/oval cells in various bunches	−	−	−	+	−	+	+	+	+	+	+	+	−	+	+	+	+	+	+	*Staphylococcus aureus*
Small pink shiny smooth colonies on MacConkey agar (MA)	Gram-negative short rods, singles and some in groups	−	+	−	+	−	−	+	−	+	−	−	−	−	+	±	+	+	+	+	*Escherichia coli*
Flat large spreading colonies with dark-blue colouration on nutrient agar (NA)	Gram-negative, single short rods and some short chains	−	+	+	−	+	−	−	−	−	−	−	−	−	+	−	−	−	+	−	*Pseudomonas aeruginosa*

*Identification of bacteria isolated from unsealed drug containers.

+, positive; −, negative; MA, MacConkey agar; NA, nutrient agar; VP, Voges–Proskauer.

**Table 4.** Identification of fungi isolated from unsealed drug containers

**Macroscopy/colony morphology**				**Microscopy**	
**Nature of colony**	**Reverse side**	**Texture**	**Nature of growth**	**Characteristics**	**Organism**
Whitish cream-coloured, pasty and smooth SDA	White	Creamy	Rapid	Septate pseudohyphae with clusters of round blastospores at the septa and large thick-walled terminal clamydospores, **positive for germ tube test**	*Candida albicans*
Woolly, whitish yellow edge with a covering of brown, which then turns dark brown to black	White to yellow	Woolly	Rapid	Septate hyphae with unbranched conidiophores arising from specialized foot cell. The conidiosphores are enlarged at the tip, forming rounded vesicles, which are covered with flask shapes that have chains of smooth dark brown conidia	*Aspergillus niger*

Table 5 shows the bacterial isolates. The most common bacterial isolates were *S. aureus* (51.8 %), followed by *E. coli* and *P. aeruginosa*. The Uchems pharmacy had the highest proportion of bacterial isolates (37 %), followed by the Stepwise pharmacy (22.2 %), while the Roseline Health Clinic had the lowest proportion (7.4 %)

**Table 5.** Types of bacteria isolated from unsealed drug containers in relation to the pharmacies and healthcare facilities

Bacteria	No. of samples contaminated (%)	Pharmacies			Primary healthcare facilities	
		Uchems	Silver	Stepwise	Ihiagwa Health Centre	Roseline Health Clinic
*Staphylococcus aureus*	14 (51.8)	5 (35.7)	2 (14.3)	3 (21.4)	3 (21.4)	1 (7.1)
*Escherichia coli*	8 (29.6)	3 (37.5)	2 (25.0)	2 (25.0)	1 (12.5)	0 (0.0)
*Pseudomonas aeruginosa*	5 (18.5)	2 (40.0)	1 (20.0)	1 (20.0)	0 (0.0)	1 (20.0)
Total	27 (100)	10 (37.0)	5 (18.5)	6 (22.2)	4 (14.8)	2 (7.4)

%, percentage.

Table 6 shows the fungal isolates. The fungal contaminants included *C. albicans* (73.3 %) and *A. niger* (26.7 %). The Uchems pharmacy had the highest proportion of fungal isolates (40.5 %) and the Roseline Health Clinic had the lowest proportion (6.7 %).

**Table 6.** Types of fungi isolated from unsealed drug containers in relation to the pharmacies and healthcare facilities

Fungi	No. of samples contaminated (%)	Pharmacies			Primary healthcare facilities	
		Uchems	Silver	Stepwise	Ihiagwa Health Centre	Roseline Health Clinic
*Candida albicans*	11 (73.3)	4 (36.4)	2 (18.2)	2 (18.2)	2 (18.2)	1 (9.1)
*Aspergillus niger*	4 (26.7)	2 (50.0)	0 (0.0)	1 (25.0)	1 (25.0)	0 (0.0)
Total	15 (100)	6 (40.5)	2 (13.3)	3 (20.0)	3 (20.0)	1 (6.7)

%, percentage.

## Discussion, conclusion and recommendations

### Discussion

The presence of micro-organisms in unsealed drug containers is a significant concern, as it can lead to compromised drug quality and safety. The study revealed that a significant proportion of unsealed drug samples (84%) were contaminated with micro-organisms. This finding underscores the critical need for robust quality control measures to prevent microbial contamination during drug manufacturing, packaging and distribution [10]. The most commonly isolated micro-organisms from this study included bacteria and fungi. The contamination of pharmaceutical products with micro-organisms is a critical concern for public health [11]. Unsealed drug containers, in particular, are susceptible to microbial contamination during storage and transportation [6]. This high prevalence of contamination is consistent with previous findings that highlight the vulnerability of unsealed drugs to microbial intrusion [1].

The most common contaminants present were bacteria, accounting for 27 out of 50 samples that tested positive. The identified bacteria, including *S. aureus*, *P*. *aeruginosa* and *E. coli*, have the potential to cause infections in patients who have been exposed to contaminated drugs. Fungal contaminants, such as *C. albicans* and *A. niger*, can also lead to adverse reactions and other health complications. Accurate identification of these bacterial isolates is crucial in assessing the potential risks associated with drug contamination [1]. This study utilized morphological and biochemical tests to identify *S. aureus*, *E. coli* and *P.s aeruginosa*. While these methods are valuable, they may have limitations in identifying certain bacterial species. Advanced molecular techniques such as PCR and DNA sequencing offer a more precise means of identification [12]. However, the discovery of these pathogenic bacteria serves as a reminder of the importance of thorough quality control in pharmaceutical handling. Identifying and isolating these bacterial species is crucial, as their presence can lead to infections among individuals using contaminated medications. These findings align with previous research indicating that *S. aureus, E. coli* and *P. aeruginosa* are commonly found in contaminated pharmaceutical products [13]. The ability of these bacteria to cause harm to patients highlights the critical need for stringent quality control measures to be implemented urgently [1].

The identification of bacterial contaminants, specifically *S. aureus*, *E. coli* and *P. aeruginosa*, within unsealed pharmaceuticals raises significant concerns with regard to patient safety [14]. *S. aureus* is recognized for its capacity to cause a spectrum of infections, encompassing skin maladies, abscesses and potentially life-threatening bloodstream infections [14]. *E. coli* has been linked to gastrointestinal ailments, urinary tract infections [9] and even septicaemia in severe instances [15]. *P. aeruginosa* is notorious for its propensity to provoke respiratory infections, particularly in individuals with compromised immune systems [16]. The potential of these bacteria to incite infections in patients who ingest contaminated drugs underscores the paramount importance of stringent quality control measures across all phases of pharmaceutical storage and distribution [17].

*C. albicans* is a prevalent fungal pathogen implicated in various infections, particularly among immunocompromised individuals [18]. On the other hand, *A. niger* is known to produce mycotoxins and can precipitate allergic reactions and respiratory issues, especially in individuals with pre-existing pulmonary conditions [19]. The detection of these fungal contaminants in unsealed pharmaceuticals accentuates the need for rigorous quality control protocols throughout the pharmaceutical supply chain [20].

It was observed that similar bacterial and fungal species were seen across the pharmacies and healthcare facilities, showing that contamination was a problem in all of the samples. It is worth noting that there are no routine screenings of the personnel in charge of the drug storage areas in these pharmacies and healthcare facilities and there are no hand hygiene audits for the staff. The personnel dispensing the drugs/pills are auxiliary nurses who have not been properly trained. The patients that were being served from these dispensaries were mostly university students, motorbicycle riders, tricycle riders and farmers, whose low economic status may serve as a constraint on acquiring better drugs for treatment. Inadequate handling, improper repackaging and failure to adhere to good manufacturing practices during the dispensing and packaging processes have led to the microbiological contamination of pharmaceutical products as reported by El-Gawad El-Sayed Ahmed *et al*. [21].

### Conclusion

This study highlights the critical issue of microbial contamination in unsealed drugs and underscores the importance of stringent quality control measures in the wholesale and retail sectors for pharmaceutical products. The identification of potentially harmful micro-organisms in these drug sample containers emphasizes the need for improved handling and storage practices to ensure product safety and efficacy. Continuous monitoring and surveillance are essential to prevent microbial contamination and protect public health. Further research and investigations are required to explore the specific sources and mechanisms of contamination in unsealed drug products and to develop effective preventive strategies.

### Recommendations

Based on the findings of this study, the following recommendations are made.

- Pharmaceutical product sellers should implement rigorous quality control measures, such as wearing of gloves and use of sterile spoons to minimize the risk of microbial contamination during drug dispensation.
- Healthcare facilities and pharmacies should enforce proper storage and handling practices to prevent drug contamination after purchase.
- Regulatory agencies should establish and enforce standards for drug storage to ensure product safety and efficacy.
- Public awareness campaigns should educate consumers about the risks associated with unsealed drug and the importance of checking drug packaging for tampering before use.
